# Association between simultaneity of health-risk behaviours and self-rated health in Brazilian adolescents

**DOI:** 10.1371/journal.pone.0271503

**Published:** 2022-07-14

**Authors:** Alexsandra da Silva Bandeira, Giovani Firpo Del Duca, Rodrigo Sudatti Delevatti, Sofia Wolker Manta, Pablo Magno Silveira, Larissa dos Santos Leonel, Leandro F. M. Rezende, Kelly Samara Silva

**Affiliations:** 1 Department of Physical Education, School of Sports, Federal University of Santa Catarina, Florianopolis, Santa Catarina, Brazil; 2 Department of Preventive Medicine, Escola Paulista de Medicina, Universidade Federal de São Paulo, São Paulo, Brazil; Public Library of Science, UNITED KINGDOM

## Abstract

**Introduction:**

This study examined the association between simultaneity of four health-risk behaviours, namely, low levels of moderate-to-vigorous physical activity (insufficient MVPA: <420 min/week), tobacco use, alcohol consumption, and excessive television (TV)–(>2 h/d of TV viewing) and self-rated health (SRH) in Brazilian adolescents.

**Methods:**

We used data of 100,551 adolescents from the National School Health Survey, a national cross-sectional study carried out in 2015. Association between simultaneity of health risk behaviours (i.e. the ratio between observed and expected prevalence rates) and SRH was examined using logistic regression models.

**Results:**

The majority of the participants were female (51.9%) and 14 years of age (50.6%), and 26% of the participants’ SRH ranged from ’average’ to ’extremely poor’. Those who engaged in the following combinations of health-risk behaviours had higher odds of worse SRH than their healthier counterparts: insufficient MVPA and tobacco use (odds ratio—OR: 2.0, 95% confidence interval [CI]: 1.4 to 3.0); insufficient MVPA and alcohol consumption (OR: 1.6, 95%CI: 1.3 to 1.9); insufficient MVPA and >2 h/day of TV viewing (OR: 1.3, 95%CI: 1.1 to 1.6); insufficient MVPA, tobacco use and alcohol consumption (OR: 2.1, 95%CI: 1.7, to 2.7); and insufficient MVPA, alcohol consumption and >2 h/day of TV viewing (OR: 1.6, 95%CI: 1.4 to 2.0).

**Conclusions:**

Insufficient MVPA, alcohol consumption, and other health-risk behaviours were associated with worse SRH in Brazilian adolescents.

## Introduction

Adolescence is a developmental stage during which one undergoes physical, psychological, and social changes, and the adoption of health-related behaviours during this period may persist into adulthood [1]. Engagement in health-risk behaviours during adolescence can negatively affect self-rated health (SRH) [2], which is an important marker of current and future health-related outcomes and quality of life [3,4].

SRH is a complex and subjective construct, primarily because it encompasses physical, emotional, and social dimensions; and this may be an important indicator of adolescent health status [5]. Poor SRH is associated with poor academic performance [6], mental health problems [3], and diseases [4]. Health-risk behaviours such as alcohol consumption, smoking, insufficient moderate-to-vigorous physical activity (MVPA), and excessive television (TV)-viewing are strongly related to poor SRH among adolescents [3,7]. However, these findings have been obtained in mutually-adjusted associations between isolated health-risk behaviours and SRH.

Despite the importance of examining the simultaneity of health-risk behaviours, few studies have investigated its association with SRH in adolescents, particularly from low and middle-income countries [8,9]. Therefore, examining the association between simultaneity of health-risk behaviours and SRH in different cultural and social contexts may help to inform education and health policies that seek to promote healthy habits in the school environment [10]. Consequently, in the long term, it may be feasible to reduce the population prevalence rate of health-risk behaviours among adolescents and thereby contribute to the improvement of their SRH [11].

Based on the aforementioned gaps in the literature, we examined the association between simultaneity of health-risk behaviours (i.e. tobacco use, alcohol consumption, insufficient MVPA, excessive TV-viewing time) and SRH in Brazilian adolescents.

## Materials and methods

### Design and participants

We used the data collected as a part of a large representative health survey conducted by the National Adolescent School-based Health Survey (PeNSE) in 2015. The PeNSE enrolled adolescents attending the ninth year of elementary school across the 26 state capitals and the Federal District of Brazil. The sample criteria were confined to include regular students who attended public or private schools in which at least 15 students were enrolled in the current academic year [12]. Data collection was undertaken between April and September 2015; the participants responded to a self-administered questionnaire using smartphones. Further details about the methodology of the PeNSE 2015 are available elsewhere [12].

The sampling strata were the 26 state capitals and the Federal District of Brazil. A cluster sampling was used, and the school and classes served as the primary and secondary sampling units, respectively. In other words, schools were initially stratified based on their geographical location and administrative dependence, and the classes that these schools entailed were selected. All the students who belonged to the selected classes were invited to respond to the questionnaires. More details about the sampling procedure and other methodological issues that the PeNSE 2015 entailed are available elsewhere [12].

### Self-rated health

SRH was measured using a single question based on the Brazilian adaptation of the Global School-Based Student Health Survey [12]: "*How would you rate your health status*?" with five response options dichotomised as ’worse SRH’ (extremely poor, poor, and average) or ’good SRH’ (good and very good) for analytic purposes. Considering that the response option "*average*" was included in the ’worse SHR’, it should be highlighted that this category means worse than good health.

### Health-risk behaviours and covariates

Health-risk behaviours were measured using a standardised questionnaire [13] that assessed MVPA, tobacco, alcohol, and TV viewing. The following question assessed the MVPA: *In the past seven days*, *how many days did you practice physical activity for at least 60 minutes (add up all the time you spent on any type of physical activity each day)*? with answers ranging from none to seven days a week. The insufficient MVPA was classified as less than 420 min/week. Two questions measured the use of tobacco and alcohol in the last 30 days, classified as a risk behaviour if the students answered "*yes*" (meaning any consumption). The TV-viewing time was evaluated using the following question: *On a regular weekday*, *how much time do you spend watching TV*? classified as a risk when the students answered more than two hours per day.

Covariates included sex, age (11–13 years, 14–15 years, and 16–19 years), and maternal educational level (i.e. ≤ seven years, eight to ten years, 11 years, and ≥ 12 years).

### Statistical analysis

SRH and health-risk behaviours were analysed in terms of their prevalence and the corresponding 95% confidence intervals (CI). The sample design (strata, conglomerates, and sample weights) was taken into consideration when descriptive and inferential analyses were performed.

In order to examine the simultaneity of health-risk behaviours, we analysed their observed (i.e. the number of participants who did not meet the guideline levels for each health-risk behaviour/the total number of participants) and expected (i.e. the proportion of participants who did not meet a specific guideline × the proportion of participants who met the guidelines for the remaining health-risk behaviours) prevalence. Health-risk behaviours were considered to be clustered when the observed prevalence of the simultaneity of behaviours was higher than the expected prevalence for the respective simultaneity [14]. In other words, ratios between the observed and expected (O/E) prevalence rates that were greater than one were indicative of clustering; accordingly, only these clusters were examined with regard to their association with SRH.

Multivariable logistic regression models were used to examine the associations between the simultaneity of health-risk behaviours and SRH, adjusted for sex, age, and maternal education level; the category ’no health-risk behaviours’ served as the reference group. We tested for interaction by including the cross-term of health-risk behaviours and sex into the model, but no evidence of interaction of the multiplicative scale was observed. Therefore, multivariable models were performed considering the entire sample and adjusted for covariates.

We applied the Bonferroni correction, a multiple comparisons correction used when several statistical tests are conducted simultaneously [15]. In other words, the level of significance (i.e. 0.05) is divided by the number of associations that are to be tested (i.e. 16). Therefore, results that entailed *p*-values that were lower than 0.003 were considered to be statistically significant. The responses of all the participants who had provided complete data about health-risk behaviours, covariates, and SRH were subjected to statistical analyses (total number of missing responses: 1.46%). All statistical analyses were conducted using version 13.0 of Stata.

### Ethical considerations

The PeNSE 2015 was approved by the National Committee of Ethics in Research (CONEP no. 1,006,467, dated March 30, 2015), which regulates and approves health research that involves human participants. Free informed verbal consent was obtained by all participants, in which they marked be aware of their participation in the research. Their parent’s or responsible person’s consent was waived for this study due to data collection logistics; that is, students filled out the online consent and then responded to the questionnaire. The students could withdraw from the research at any moment. Additional information about the PeNSE methods and sampling procedures is published elsewhere [16].

## Results

A total of 100,551 adolescents were included in our study; a majority of the participants were girls (51.9%) and 14 years of age (50.6%). About 26% of the students self-reported their health status (SRH) from ’average’ to ’extremely poor’. The students who had worse SRH were more likely girls, 16 to 19 years, and had lower maternal education than those with good SRH (Table 1). Students with worse SRH were also more likely to use tobacco and consume alcohol in the previous 30 days, had insufficient MVPA and viewed TV for > 2 hours per day than those with good SRH (Table 1).

**Table 1:** Sociodemographic and Behavioural characteristics in Brazilian adolescents according self-rated health. National Adolescent School-based Health Survey (PeNSE). Brazil 2015 (n = 100,551).

Variables	Worse SRH				Good SRH			
	N	% ^a^	95% CI		N	% ^a^	95% CI	
**Sex**								
Girls	17,315	60.0	58.9	61.1	34,868	48.4	47.6	49.1
Boys	11,317	40.0	38.9	41.1	37,051	51.6	50.9	52.4
**Age group**								
11–13 years	4,630	17.1	16.0	18.3	12,424	18.7	17.6	19.9
14–15 years	20,318	70.7	69.4	71.9	51,124	70.9	69.7	72.0
16–19 years	3,684	12.2	11.4	13.0	8,371	10.4	9.8	11.0
**Maternal education**								
≤ 7 years	7,130	36.0	34.5	37.5	16,298	33.0	31.8	34.2
8–10 years	3,628	17.7	16.7	18.6	8,534	17.0	16.3	17.7
11 years	5,008	24.2	23.1	25.4	12,727	24.8	23.9	25.6
≥ 12 years	5,829	22.1	20.8	23.5	16,663	25.3	23.8	26.8
**Tobacco use**								
Yes	2,058	8.0	7.3	8.8	3,236	4.6	4.3	5.0
No	26,574	92.0	91.1	92.6	68,683	95.4	94.9	95.6
**Alcohol consumption**								
Yes	7,535	28.8	27.7	29.8	14,773	22.0	21.3	22.6
No	21,097	71.2	70.1	72.2	57,146	78.0	77.3	78.6
**MVPA**								
< 420 min/wk	26,536	93.3	92.7	93.8	65,282	91.0	90.6	91.4
≥ 420 min/wk	2,096	6.7	6.2	7.3	6,637	9.0	8.6	9.4
**Excessive TV time**								
> 2 h/d	17,302	62.1	60.9	63.3	41,169	59.0	57.9	59.9
≤ 2 h/d	11,330	37.9	36.6	39.0	30,750	41.0	40.0	42.0

SRH: Self-rated health; MVPA: Moderate to vigorous physical activity; Variable with the largest number of missing data: SRH (n = 754; 0.7%)

^a^ Weighted proportion; SHR had five response options dichotomised as ’worse SRH’ (extremely poor, poor, and average) or ’good SRH’ (good and very good). Considering that the response option "average" was included in the "worse SHR", it should be highlighted that this category means worse than good health.

Table 2 shows the observed and expected prevalence rates, and the O/E ratios for the simultaneity of health-risk behaviours in Brazilian students. The isolated health-risk behaviours that had an observed prevalence rate that was higher than the expected prevalence rates were alcohol consumption (O/E = 11.18, 95% CI: 10.40 to 12.00) and insufficient MVPA (O/E = 67.07, 95% CI: 66.30–67.80). The following combinations of health-risk behaviours had observed prevalence rates higher than the expected prevalence rates: tobacco use and insufficient MVPA (O/E = 9.89, 95% CI: 9.00 to 10.83), alcohol consumption and insufficient MVPA (O/E = 3.83, 95% CI: 3.73– to 3.93), insufficient MVPA and >2 hours per day of TV-viewing time (O/E = 35.43, 95% CI: 35.10 to 35.80), tobacco use, alcohol consumption, and insufficient MVPA (O/E = 8.00, 95% CI: 7.60 to 8.40), alcohol consumption, insufficient MVPA, and >2 hours per day of TV-viewing time (O/E = 2.57, 95% CI: 2.52 to 2.62).

**Table 2:** Simultaneity of health-risk behaviours in Brazilian students. National Adolescent School-based Health Survey (PeNSE). Brazil, 2015 (n = 100,551).

Number of health-risk behaviours	Presence of health-risk behaviours				n*	O ^a^ (%)	E (%)	O/E ^b^ (95% CI)
	Tobacco	Alcohol	MVPA	TV time				
**0**	-	-	-	-	2,855	0.30	0.62	4.52 (4.36 to 4.69)
**1**	+	-	-	-	48	0.00	2.17	0.02 (0.02 to 0.03)
	-	+	-	-	716	0.10	0.06	11.18 (10.40 to 12.00)
	-	-	+	-	30,690	28.80	0.44	67.07 (66.30 to 67.80)
	-	-	-	+	3,562	0.34	11.03	0.32 (0.31 to 0.33)
**2**	+	+	-	-	174	0.00	0.22	0.77 (0.67 to 0.99)
	+	-	+	-	459	0.00	0.04	9.89 (9.00 to 10.83)
	+	-	-	+	77	0.06	38.80	0.00 (0.00 to 0.00)
	-	+	+	-	6,164	0.63	1.57	3.83 (3.73 to 3.93)
	**-**	**+**	**-**	**+**	1,095	0.12	8.00	0.13 (0.12 to 0.14)
	**-**	**-**	**+**	**+**	40,480	40.11	1.12	35.43 (35.10 to 35.80)
**3**	+	+	+	-	1,306	0.12	0.16	8.00 (7.60 to 8.40)
	+	+	-	+	279	0.02	0.81	0.34 (0.30 to 0.38)
	+	-	+	+	632	0.10	28.13	0.02 (0.02 to 0.02)
	-	+	+	+	10,343	11.30	3.93	2.57 (2.52 to 2.62)
**4**	**+**	**+**	**+**	**+**	2,395	0.30	2.85	0.82 (0.80 to 0.90)

+: Presence of risk factor / −: Absence of risk factor; O

^a^: Observed proportion weighted; E: Expected; O/E

^b^: Observed prevalence (n) / expected prevalence (n); MVPA: Less than 420 minutes/week; TV time: More than 2 hours/day.

*: Number of adolescents in each group.

**Fig 1:**
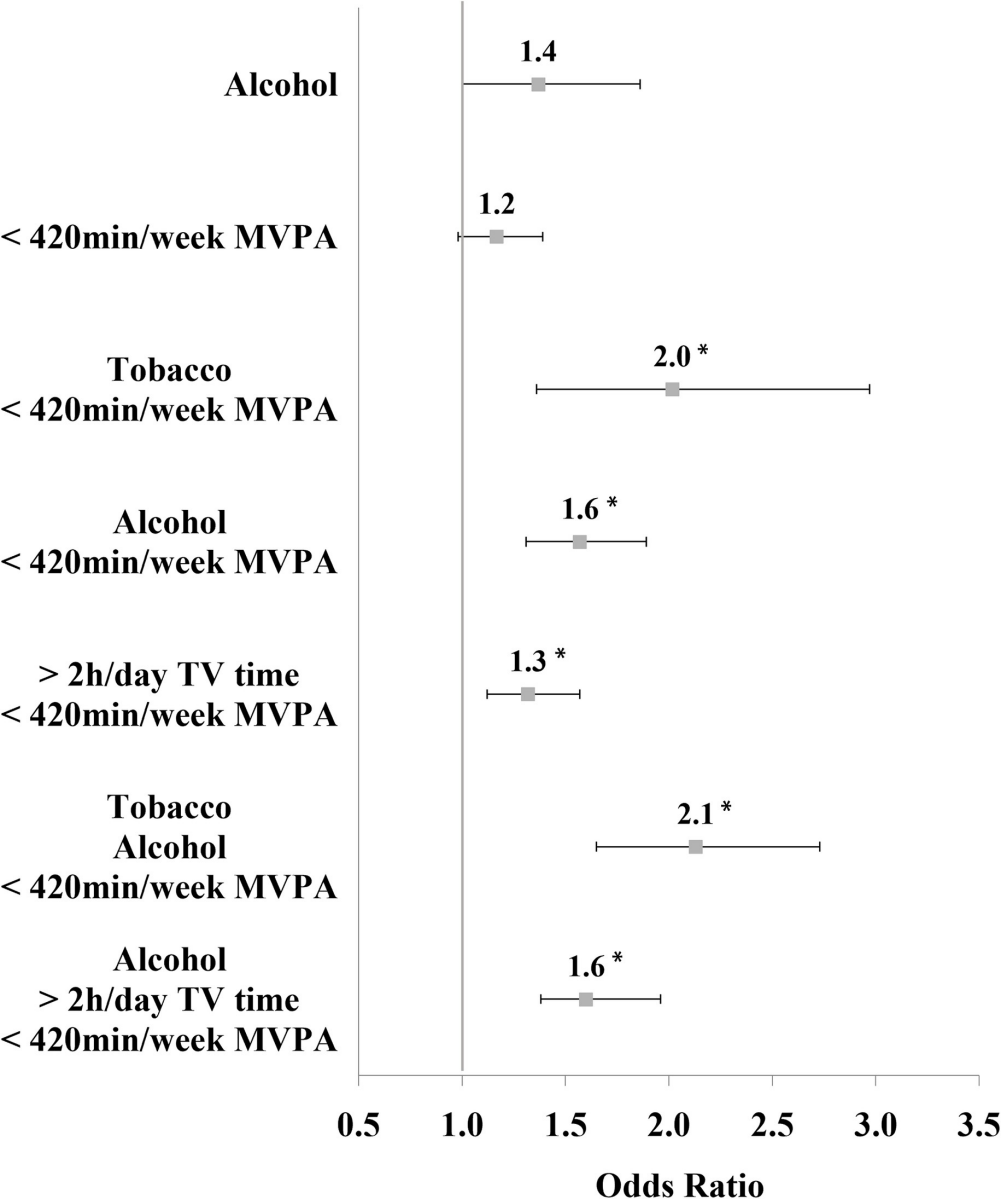
Association of simultaneity of health-risk behaviours with worse self-rated health in Brazilian adolescents. National Adolescent School-based Health Survey 2015 (PeNSE). Note: Category "no risk behaviours" was used as a referent; MVPA: Moderate to vigorous physical activity; Odds ratio is adjusted for sex, age and maternal education. *: Represents a statistically significant p-value <0.003.

The O/E ratios greater than one were subjected to the analyses examining the crude and adjusted associations between health-risk behaviours and worse SRH (Fig 1). The results of the adjusted model revealed that the students who reported the following combinations of health-risk behaviours had higher odds of worse SRH than those who did not engage in health-risk behaviours: tobacco use and insufficient MVPA (odds ratio [OR] = 2.0, 95% CI: 1.4 to 3.0), alcohol consumption and insufficient MVPA (OR = 1.6, 95% CI: 1.3 to 1.9), >2 hours per day of TV-viewing and insufficient MVPA (OR = 1.3, 95% CI: 1.1 to 1.6), tobacco use, alcohol consumption, and insufficient MVPA (OR = 2.1, 95% CI: 1.7 to 2.7), alcohol consumption, >2 hours per day of TV-viewing, and insufficient MVPA (OR = 1.6, 95% CI: 1.4 to 2.0).

## Discussion

In the present study, we analysed a large and representative sample of Brazilian adolescents. We found that those who reported a combination of insufficient MVPA and either one (tobacco use, alcohol consumption, or excessive TV-viewing) or two (tobacco use and alcohol consumption or alcohol consumption and excessive TV-viewing) health-risk behaviours had higher odds of worse SRH. Of note, insufficient MVPA and alcohol consumption were featured in all the emergent combinations of health-risk behaviours associated with poor SRH.

Adolescents who did not engage in the recommended levels of MVPA and either used tobacco, consumed alcohol, or watched TV for more than 2 hours per day were more likely to have worse SRH. These results are in agreement with previous studies [17,18]. For example, Craig and colleagues analysed several factors (including health-risk behaviours) that directly or indirectly influence the SRH of Australian adolescents [17]. They found that physical activity was directly associated with better SRH and indirectly associated with SRH through mental health- and vitality-related variables. In other words, students with higher physical activity levels tended to have better mental health and greater vitality and, consequently, better SRH [17]. Since the adoption of even one healthy behaviour may facilitate engagement in other health-promoting behaviours [3], the promotion of MVPA may foster a healthier lifestyle and contribute to better SRH in adolescents.

Furthermore, we found that the simultaneity of alcohol consumption and other risk behaviours (e.g., insufficient MVPA, tobacco use, and excessive TV-viewing) was also associated with worse SRH in adolescents. Although the sale of alcoholic beverages to adolescents who are less than 18 years of age is prohibited in Brazil, alcohol consumption is prevalent among this age group [19,20] due to parental approval [21], easy access to alcoholic beverages [22], and the need to escape from stress, anxiety, and familial and school-related problems [23]. However, it is well documented that alcohol consumption negatively influences cognitive, emotional, and social development during adolescence and adulthood [24,25]. Alcohol consumption affects adolescent behaviours and may lead to violence and emotional changes [26]; in addition, it may lead to the use of other drugs, such as tobacco smoking [27]. Given the simultaneity of insufficient MVPA and excessive TV viewing, adolescents who engage in these health-risk behaviours may be exposed to advertisements that encourage the consumption of alcoholic beverages [28]. In this regard, strategies that reduce alcohol consumption among adolescents may facilitate behaviour change and consequently improve SRH.

About the number of health-risk behaviours, engagement in a single health-risk behaviour (i.e., alcohol consumption or insufficient MVPA) was not associated with worse SRH in adolescents. Engagement in numerous healthy behaviours may attenuate the otherwise harmful effects of a given health-risk behaviour [29,30]. However, the simultaneous presence of healthy behaviours in adolescents was relatively low in the present study (1.3%). Moreover, we observed that simultaneities that entailed two or three health-risk behaviours were associated with worse SRH, even though the magnitudes of their relationships were similar. This finding reinforces the contention that different cultural, social, and intrapersonal factors may influence the association between the simultaneity of health-risk behaviours and adolescent health [14,29]. These associations may vary depending on the contexts and developmental stages within which they are analysed [5,31].

### Study limitations and strengths

The strength of the present study is that it explored the association between the simultaneity of several health-risk behaviours and SRH in Brazilian adolescents who belong to a middle-income country. Moreover, the present study used population-based data collected using probabilistic sampling and adequate methodological rigour; therefore, the study’s results can be generalised to the target population. However, some of the limitations of the present study are also noteworthy. First, due to the cross-sectional design of the present study, inferences about the causality of the emergent associations cannot be drawn. Second, health behaviours were self-reported measures, and thus measurement error may have occurred. Third, although TV viewing is an important predictor of health outcomes, especially in low and middle-income countries, our study only assessed TV time. However, different screen devices, contents, and types might have a different impact on SRH, for instance, cell phone use and gaming [32]. Finally, although different health-risk behaviours were analysed in the present study, other health-risk behaviours (e.g., unhealthy diet) may also influence adolescents’ SRH.

## Conclusions

In conclusion, our results showed that the simultaneity of health-risk behaviours was associated with worse SRH in Brazilian adolescents. Insufficient MVPA combined with alcohol consumption and other health-risk behaviours (e.g., tobacco use and excessive TV viewing) was associated with worse SRH in adolescents. These findings may be useful to those who design school-based interventions to promote healthy behaviours and improve SRH in adolescents.
